# The metabolomics workbench file status website: a metadata repository promoting FAIR principles of metabolomics data

**DOI:** 10.1186/s12859-023-05423-9

**Published:** 2023-07-24

**Authors:** Christian D. Powell, Hunter N. B. Moseley

**Affiliations:** 1 Department of Computer Science (Data Science Program), University of Kentucky, Lexington, KY 40506 USA; 2 Markey Cancer Center, University of Kentucky, Lexington, KY 40506 USA; 3 Superfund Research Center, University of Kentucky, Lexington, KY 40506 USA; 4 Department of Molecular and Cellular Biochemistry, University of Kentucky, Lexington, KY 40506 USA; 5 Institute for Biomedical Informatics, University of Kentucky, Lexington, KY 40506 USA

**Keywords:** Metabolomics Workbench, Validation, mwtab, FAIR, Metadata repository, Website

## Abstract

**Background:**

An updated version of the mwtab Python package for programmatic access to the Metabolomics Workbench (MetabolomicsWB) data repository was released at the beginning of 2021. Along with updating the package to match the changes to MetabolomicsWB’s ‘mwTab’ file format specification and enhancing the package’s functionality, the included validation facilities were used to detect and catalog file inconsistencies and errors across all publicly available datasets in MetabolomicsWB.

**Results:**

The MetabolomicsWB File Status website was developed to provide continuous validation of MetabolomicsWB data files and a useful interface to all found inconsistencies and errors. This list of detectable issues/errors include format parsing errors, format compliance issues, access problems via MetabolomicsWB’s REST interface, and other small inconsistencies that can hinder reusability. The website uses the mwtab Python package to pull down and validate each available analysis file and then generates an html report. The website is updated on a weekly basis. Moreover, the Python website design utilizes GitHub and GitHub.io, providing an easy to replicate template for implementing other metadata, virtual, and meta- repositories.

**Conclusions:**

The MetabolomicsWB File Status website provides a metadata repository of validation metadata to promote the FAIR use of existing metabolomics datasets from the MetabolomicsWB data repository.

## Background

In 2006, the Congress of the United States of America passed the National Institutes of Health (NIH) Reform Act of 2006 to reauthorize and reorganize the NIH, which also established the NIH Common Fund to support cross-cutting, trans-NIH programs which are involved in two or more of the NIH’s Institutes or Centers. Today, the Common Fund consists of more than 30 active projects and has funded more than 50 projects in total [1]. One currently funded project is the Metabolomics project to inform basic, translational, and clinical research. The Common Fund’s Metabolomics project established the Metabolomics Workbench (MetabolomicsWB) as a longstanding, public repository for national and international metabolomics data and metadata [2]. Today, MetabolomicsWB is host to more than 2200 studies with over 3600 individual metabolomics analyses currently available.

In 2018, the mwtab Python package was initially released with the intent of providing a ‘pythonic’ means of access and manipulation of data and metadata hosted on MetabolomicsWB [3]. The package was developed with the intention of promoting the FAIR principles of data to be findable, accessible, interoperable, and reusable [4, 5]. Along with the release of the mwtab Python package, the package was used to find a number of inconsistencies between the mwTab file format specification and actual mwTab data files hosted by MetabolomicsWB.

With the release of the 1.1.0 version of the mwtab Python package [6], the MetabolomicsWB file validation functionality of the package was greatly improved and updated to follow the latest mwTab file format specification. The mwtab package was again used to find formatting errors in mwTab files, uncovering that a majority of the datafiles hosted on MetabolomicsWB have some degree of inconsistency to their file format specification.

We developed the MetabolomicsWB File Status website to inform curators maintaining MetabolomicsWB, researchers depositing datasets, and researchers reusing MetabolomicsWB datasets, of possible issues with specific datasets. The website uses the mwtab Python package to provide a simple way to determine which analysis files in the MetabolomicsWB contain file formatting errors, inconsistencies, or are simply unavailable through MetabolomicsWB’s REST interface.

### Implementation

The Metabolomics Workbench (MetabolomicsWB) File Status website is available through GitHub Pages [7]. All scripts and files used to generate the website are made open-source and are publicly available within the GitHub repository hosting the website [8]. Installation instructions are included within the GitHub repository. This package only directly depends on the mwtab package [6, 9].

### The Metabolomics Workbench file status website

**Fig. 1:**
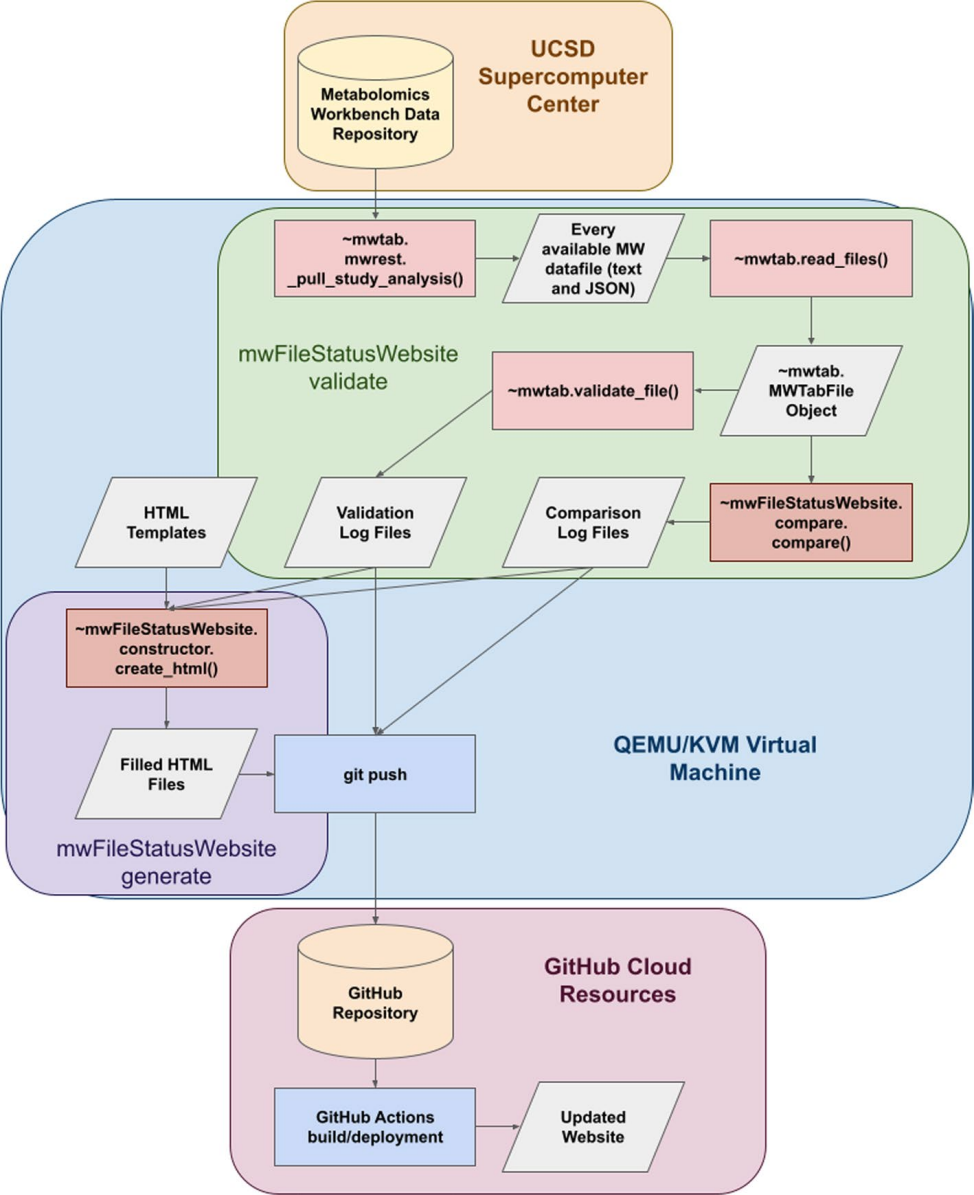
Flowchart of Build Process for Updating the Metabolomics Workbench File Status Website. A single bash script containing all the required commands to update the MetabolomicsWB File Status website is run on a QEMU/KVM virtual machine hosted on a Linux server to update the MetabolomicsWB File Status website. The script first creates a virtual environment and then executes multiple commands to download, generate, and push all the required files to a GitHub repository and relies on functions from the mwtab and mwFileStatusWebsite Python3 packages. The GitHub repository then performs building and deployment actions to update the GitHub.io based website. The first command executed is the “mwFileStatusWebsite validate” command which involves using the ~ mwtab.mwrest._pull_study_analysis() function to pull down and save each available metabolomics dataset from the Metabolomics Workbench data repository hosted by the UC San Diego Supercomputer Center. The data files are saved locally in both their mwTab (plain text) and JavaScript Object Notation (JSON) formats. Next the ~ mwtab.read_files() function is used to convert the local files into ~ mwtab.MWTabFile objects. The objects are then used by the ~ mwtab.validate_file() and ~ mwFileStatusWebsite.compare.compare() functions to generate the needed validation and comparison log files. Next, the “mwFileStatusWebsite generate” command uses the validation and comparison data, along with structured HTML templates, to form the updated HTML pages of the website. This is accomplished by using the ~ mwFileStatusWebsite.constructor.create_html() function. Next, the bash script uses git to add all the changed validation logs, comparison logs, and updated HTML pages before being committed and pushed to the remote repository hosted on GitHub. Upon being pushed, the remote GitHub repository uses GitHub’s cloud resources to trigger an action to build and deploy the updated HTML pages to the GitHub Pages hosting. The bash script finishes by deleting temporary files and the virtual environment used

The MetabolomicsWB file status website is a series of HTML pages hosted via GitHub Pages. The HTML files are regularly updated using a series of Python scripts hosted in the same GitHub repository with the GitHub Pages deployment. These scripts extensively use the mwtab Python package to collect and validate the available analysis data files from MetabolomicsWB. The scripts are executed within a QEMU/KVM virtual machine running Fedora 35 with the following allocated virtual resources: 2 “Intel Core Processor (Haswell)” CPU cores, 4 GB RAM, and 20 GB virtual disk. The virtual machine is running on a CentOS 7.2 application server with the following hardware: dual Intel(R) Xeon(R) CPU E5-2650 v4 @ 2.20 GHz processors (12 cores with hyperthreading), 256 GB RAM, 10 Gb Ethernet, 88 TB RAID6 array with 2 hot spares and dual 1 TB SSD cache. The first script to be run is the ~ mwFileStatusWebsite.validator.py script. The script uses the ~ mwtab.mwrest._pull_study_analysis() method to generate a dictionary containing a mapping of all available study IDs and their corresponding analysis IDs. The ~ mwtab.read_files() method is then used iteratively to retrieve each available analysis data file in mwTab (plain text) and JSON format. The ~ mwtab.validate_file() method is then used to generate a validation log file and collect the validation status of each analysis file. Along with validating each individual data file, the mwTab and JSON file formats are compared to ensure consistency between the two. The validation process can be run so that each individual file is read into memory and deleted once the process is complete or the process can be run so that each file is saved out to a specified output directory. It should be noted that saving out each file increases the runtime of the script and requires an increasingly large amount of storage space as more studies are added to MetabolomicsWB. Currently, saving out each individual file takes up over 3 gigabytes of storage space. The ~ mwFileStatusWebsite.compare.py script contains a series of methods for performing these comparisons based on an updated version of the comparison analysis previously performed [6]. The final script to run is the ~ mwFileStatusWebsite.generate_html.py script which contains methods for taking the collected validation and comparison statistics and inserting them into HTML templates to form the MetabolomicsWB file status website. The final templates, along with a CSS style, is then pushed to the GitHub repository and the webpage is automatically updated. Figure 1 illustrates the whole build process that updates the website.

### Updates to the mwtab Python package

**Fig. 2:**
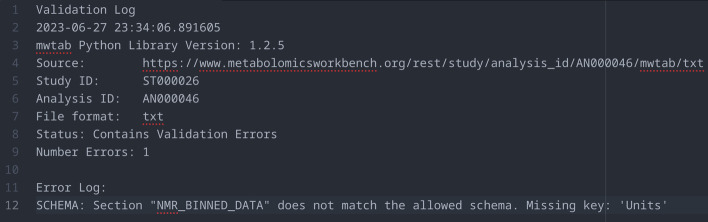
Example Validation Log Created by the mwtab Python Package. Validation logs are created by calling the ~ mwtab.validate_file() method. The validation log consists of metadata surrounding when and the conditions the validation was performed, metadata identifying the Metabolomics Workbench analysis data file the validation was performed on, and the number of validation errors collected if any

There were two major updates made to the mwtab Python3 package during the development of the MetabolomicsWB File Status website. The first update, mwtab 1.2.4, included the restructuring of validation logs generated when using the ~ mwtab.validator.validate_file() method. The method now has the option to return a structured string validation log. This change was included to allow for the generation of discrete validation logs for each available analysis data file in both mwTab (plain text) and JSON formats. Upon validation, the validation log string is captured and saved into a plaintext file (see example in Fig. 2) which is then uploaded to the GitHub repository hosting the MetabolomicsWB File Status website. Additional changes to the mwtab Python package, which occurred alongside the development of the MetabolomicsWB File Status website, were minor bug changes such as changes to the handling of blank files to allow validation logs to be generated for blank/missing datasets. The second update, mwtab 1.2.5, included changes to the ~ mwtab.mwschema and ~ mwtab.validator modules to reflect changes with the 1.5 version of the MetabolomicsWB’s mwTab file format specification.

## Results

**Fig. 3:**
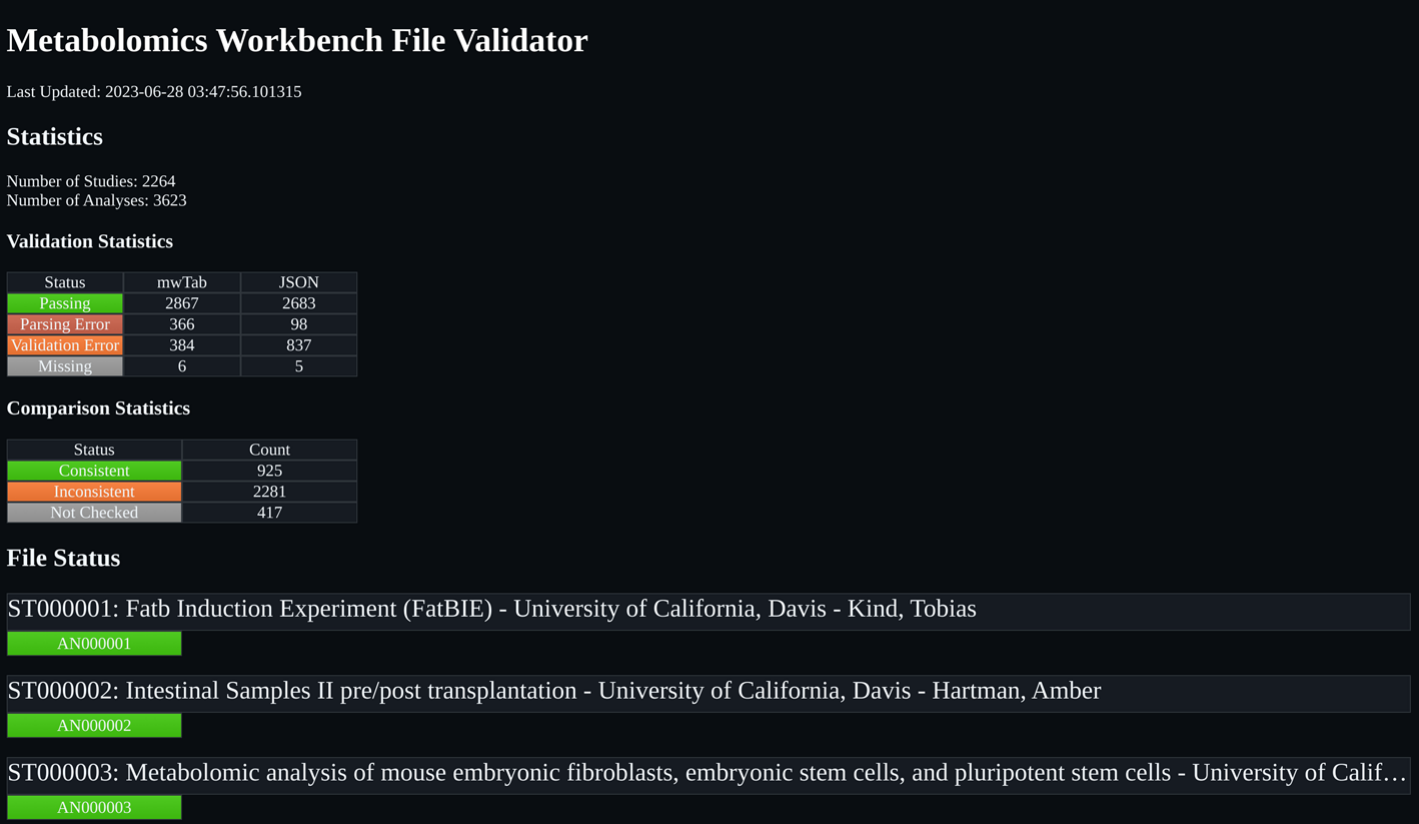
The Metabolomics Workbench File Status Website. The website uses Metabolomics Workbench’s dataset hierarchy of Studies and Analyses to logically display the file status of each file format of each analysis available from the Metabolomics Workbench data repository

The Metabolomics Workbench (MetabolomicsWB) File Status validation website is available on GitHub Pages [7] (Fig. 3). The source code for the Python scripts used to generate the webpage and validation logs for each available mwTab data file are available in the same GitHub repository that is used to host the webpage [8]. The mwtab Python library which is used to validate the analyses from MetabolomicsWB is available on GitHub [9], PyPI [10], and documentation is available on ReadTheDocs [11]. The analysis data files used for this validation were downloaded on June 25th, 2023 and the generated HTML files of the website are available for download through figshare [12] .

### File errors in Metabolomics Workbench analysis datafiles

The ~ mwtab.mwtab.validator.py module from the 1.2.5 version of the mwtab Python package was used to determine errors in both the ‘mwTab’ and JSON file formats for each available analysis data file. For the ‘mwTab’ formatted analyses, 6 files were inaccessible through MetabolomicsWB’s REST API, 366 files contained gross formatting errors preventing them from being parsed, 384 files contained minor formatting errors which were inconsistent with MetabolomicsWB’s ‘mwTab’ file format specification, and the remaining 2,867 ‘mwTab’ formatted files passed all validation. For the JSON formatted analyses, 5 files were inaccessible through MetabolomicsWB’s REST API, 98 files contained gross formatting errors preventing them from being parsed, 837 files contained minor formatting errors which were inconsistent with MetabolomicsWB’s ‘mwTab’ file format specification, and the remaining 2,683 JSON formatted files passed all validation.  These results are summarized in Table 1.

**Table 1:** File status for available analyses from Metabolomics Workbench

Status\file format	mwTab	JSON
Passing	2,867	2,683
Parsing error	366	98
Validation error	384	837
Missing	6	5

File status for each file format of each analysis available from the Metabolomics Workbench data repository. These results were generated on June 28th, 2023

Multiple of the analysis files from the ST002555 study standout as a unique issue for the “Missing” files error category. The study contains a total of 4 individual analysis files, 3 of which are inaccessible via MetabolomicsWB’s REST interface in both their mwTab and JSON file formats. However, all 4 of the analyses for study ST002555 are accessible through the main MetabolomicsWB website. The first analysis file AN004207 contains the combined experimental data for all the analyses and is available in both the mwTab and JSON formats from the MetabolomicsWB website and through the REST interface. The analysis files AN004208 to AN004210 from the MetabolomicsWB website contains metadata describing three additional mass spectroscopy methods used to acquire the experimental data, but lack the actual experimental data. Since these analysis files are inaccessible through the REST interface, they have been marked as “Missing”. But these analysis files are likely missing from the REST interface, because they lack any experimental data, thus breaking the mwTab format specification.

### File consistency in Metabolomics Workbench analysis data files

The ~ mwFileStatusWebsite.mwFileStatusWebsite.compare.py module of the 1.0.0 version of the mwFileStatusWebsite Python3 package was used to compare the ‘mwTab’ (plain text with tab separation) and JSON formatted data files for each available analysis. Of the 3623 available analyses, 925 analyses had consistent ‘mwTab’ and JSON files, 2281 analyses had inconsistent ‘mwTab’ and JSON files, and 417 analyses could not be compared due to one or more of the file formats being unparsable or missing (Table 2). While mwTab-JSON inconsistencies are likely due to errors in conversion software utilized by MetabolomicsWB, missing and unparsable files are likely due to text conversion errors and/or deposition software errors during deposition curation. Development of separate validations are required to catch these types of errors in what is expected to be a near-automated curation and conversion process.

**Table 2:** Statistics for the comparison of the Metabolomics Workbench ‘mwtab’ and JSON formatted data files

Status	Count
Consistent	925
Inconsistent	2,281
Not checked	417

Comparison statistics for the comparison of the ‘mwtab’ (plain text) and JSON formatted data files of each analysis data file available from the Metabolomics Workbench data repository. These results were generated on June 28th, 2023

## Discussion

First, a metadata repository is a repository type which provides additional data to enhance a pre-existing repository. For example, the Metabolomics Workbench (MetabolomicsWB) File Status website is technically a metadata repository that provides additional validation metadata to MetabolomicsWB analyses. Its implementation cleanly adds a GitHub-hosted Web User Interface (UI) to the validation metadata generated by the Python mwtab package. This easy-to-implement design can be duplicated for other metadata repositories as well as for virtual and meta repositories. Second, a virtual repository is a repository type which only provides access to pre-existing data without adding novel data. An example of a virtual repository is Metabolome Exchange which provides access to datasets from four different metabolomics data repositories [13]. Third, by combining both concepts and including extra data, this approach could be used to create a meta-repository, which overlays additional metadata onto a virtual repository that points to datasets, but also provides additional data access. Validated MetabolomicsWB datafiles are intentionally not provided by the MetabolomicsWB File Status website. Providing such files would shift the website from being a metadata repository to being a meta-repository. The MDACC Standardized Data Metabolomics Workbench Tool is a current example of a meta-repository which provides additional functionality allowing users to view study/analysis metadata from MetabolomicsWB and download the metabolite data section separately [14].

Prior work in validating public metabolomics datasets includes our mwtab Python package [6] that provides validation of mwTab-formatted datasets in MetabolomicsWB and the ISA-API Python library [15] that provides validation of deposited datasets into MetaboLights [16], a sister European metabolomics repository that utilizes the ISA-Tab format for deposition. Developing standalone software libraries and packages is a natural start for improving the FAIRness of public repositories, but requires a higher level of computational expertise to use. Providing these validations through web portals makes such validation tools more broadly and easily accessible. But implementing and maintaining these web portals require continual computational and labor resources. To minimize these requirements, our metadata repository implementation approach is very efficient, taking roughly one hour to update each week within a QEMU/KVM Linux virtual machine. Most of this time is somewhat idle, since we set a 1 s delay between REST pulls from MetabolomicsWB to minimize any stress on their servers and internet infrastructure. This delay is pragmatic, since currently over 3 GB of analysis files are being pulled from the MetabolomicsWB REST interface. This also factored into our decision to update the metadata repository weekly versus daily, since these files are not expected to change often making it hard to justify the daily use of network resources. If MetabolomicsWB provided a file checksum through their REST interface, file download could be skipped if checksums did not change from prior pulls, greatly reducing both network traffic and re-validation of analysis files that have not changed. While our virtualized implementation is hosted on a local server, it could be easily hosted on one of the major cloud computing services with a scheduler that periodically launches a virtual machine instance for execution and then stops the instance to minimize the cost, i.e. definitely less than $10 US dollars per year. Also, the lightweight implementation of the website utilizes the free web hosting and repository services provided by GitHub. Since all validations are automated, our implementation approach would be ideal for efficiently implementing and maintaining specialized public meta-repositories for specific scientific communities that utilize a well-established scientific repository for deposition, interoperability, and access functionality. Such meta-repositories are easier to implement and maintain than alternative full duplicate repositories like the PDB-REDO Database [17], which contains re-analyzed versions of 3D macromolecular structure entries in the worldwide Protein Data Bank [18].

## Conclusions

In developing the Metabolomics Workbench (MetabolomicsWB) File Status website, we aimed to enhance the FAIRness of the MetabolomicsWB data repository. The website serves as an additional access point for researchers wishing to find metabolomics datasets from MetabolomicsWB but is primarily intended for capturing and curating possible formatting errors, inconsistencies, and availability issues of individual datasets. By doing so, we hope to have enhanced the reusability of the increasing number of datasets hosted by MetabolomicsWB. The validation logs generated for both the text and JSON formatted mwTab datafiles can be used to quickly identify possible issues and the information the logs provide has already been used to quickly fix files by the MetabolomicsWB/UC San Diego team [6]. This information would also be very useful to researchers performing meta-analyses across datasets, since it could provide additional pragmatic filtering criteria, especially with respect to parsability and completeness. The MetabolomicsWB File Status website represents a small example of a metadata repository for which few exist. By adding additional metadata to existing datasets, this website, and metadata repositories in general, aim to improve the FAIR use of existing datasets. The lightweight implementation of the website, which utilizes the free web hosting and repository services provided by GitHub, can be copied and augmented for future metadata repositories.

MetabolomicsWB has recently released version 1.6 of their mwTab file format specification. The update included the requirement of multiple new fields in the Chromatography section of mass spectroscopy analyses. This inclusion of new required fields presents a problem for the validation of analyses which predate the update, since MetabolomicsWB does not require legacy submissions to be updated with each version change of their mwTab file format specification. One possible solution to this issue is for MetabolomicsWB to make available the mwTab file format specification for each version of the specification and include a new field in the header of each data file specifying the version used at the time of release. The mwtab Python3 package could then be updated to validate each datafile based on the version of the mwTab file format specification used upon its release.

Future improvements to the website include improving the appearance of the site and functionality. GitHub Pages allows for the use of multiple different frameworks such as Bootstrap, Jeykll, and Semantic UI which could be utilized to improve the appearance and usability of the site. The addition of advanced search methods and the ability to search across study/analysis metadata should improve the usability of the website for individual users. As mentioned, the current implementation could be used for additional metabolomics data resources. Either the current website could be augmented or a similar implementation could be used to create a new website for datasets from the MetaboLights database.

## Availability and requirements

Project name: The Metabolomics Workbench File Status Website. Project home page: https://moseleybioinformaticslab.github.io/mwFileStatusWebsite/. Code repository: https://github.com/MoseleyBioinformaticsLab/mwFileStatusWebsite. Operating system(s): Platform independent. Programming language: Python3, HTML, CSS Other requirements: Python 3.5 or higher, mwtab 1.2.5 or higher, and pytest. License: Clear BDS.

## Data Availability

The datasets supporting the conclusions of this article are available in the figshare repository, [https://doi.org/10.6084/m9.figshare.19221159].

## Abbreviations

FAIR: Findable, accessible, interoperable, and reusable guiding principles of data stewardship
HTML: Hypertext markup language
JSON: Javascript Object Notation
MetabolomicsWB: Metabolomics Workbench
NIH: National Institutes of Health
REST: Representational state transfer software architectural style
